# Diverse Approaches to Assessing What Matters to Older Adults

**DOI:** 10.1093/geroni/igaa057.1937

**Published:** 2020-12-16

**Authors:** Erin Emery-Tiburcio, Robyn Golden

## Abstract

Asking older adults What Matters to them and assuring that care plans are aligned with these preferences is the cornerstone of an Age-Friendly Health System (AFHS). Health systems have struggled to identify clear ways to ask this question and meaningfully utilize the responses. Both simple and complex options for addressing this challenge have been developed at Rush University Medical Center. At Rush, nurses began asking every inpatient What Matters and placing the response on the white board in the patient’s room. Results of this practice include increased awareness of staff and significant increases in patient satisfaction. Qualitative analysis of responses yields increased awareness of patterns that the hospital can more systematically address. The Rush Center for Excellence in Aging hosts Schaalman Senior Voices, in which older adults from diverse backgrounds are given the unique opportunity to offer their perspectives on life, health and aging related to “What Matters” to them. The films have been used effectively to stimulate conversations among older adults and families in the community and in health professions courses, and with health systems executives. The Rush College of Medicine has integrated AFHS training into communication skills for medical students. Faculty introduce the 4Ms and demonstrate methods for having What Matters (WM) conversations. Students then practice WM conversations with simulated patients; some have had the opportunity to practice with real patients in preceptorships. Implications for the health system and community will be discussed as Rush builds an Age-Friendly Health Community.

